# Papillary lesions of breast – An introspect of cytomorphological features

**DOI:** 10.4103/0970-9371.66692

**Published:** 2010-01

**Authors:** D Prathiba, Shalinee Rao, Kasthuri Kshitija, Leena Dennis Joseph

**Affiliations:** Department of Pathology, Sri Ramachandra University, Chennai, India

**Keywords:** Accuracy, breast, fine needle aspiration cytology, nipple discharge, papillary lesions

## Abstract

**Background:**

True papillary lesions of the breast have a significantly high error rate on fine needle aspiration cytology (FNAC), as many other nonpapillary breast lesions exhibit overlapping features on cytosmears.

**Aim:**

To evaluate the utility of individual morphological features in offering a more precise cytodiagnosis in papillary lesions of the breast.

**Materials and Methods:**

Cytology smears reported as papillary lesions on nipple discharge / FNAC and histopathology over a period of two years were studied and correlated. A subjective assessment of morphological features, namely, the cellular yield, presence of three-dimensional papillary clusters, stromal bare nuclei, presence of cyst macrophages and cellular atypia was carried out on cytosmears.

**Results:**

Fourteen cases of papillary lesions were identified. Thirty-six per cent of the cases were found to be true positive, 43% false negative and 21% false positive with a sensitivity of 42% for papillary lesions. Eight of nine papillary lesions showed cyst macrophages. Stromal bare nuclei were seen in three of four malignant papillary lesions. However, the number of stromal bare nuclei was less compared to benign lesions. None of the malignant nonpapillary lesions showed stromal bare nuclei.

**Conclusions:**

Cytomorphological features alone are inadequate for the precise diagnosis of papillary lesions of the breast.

## Introduction

Cytological interpretation of papillary lesions of breast continues to be a difficult task. These lesions top the list of conditions in which there is a risk of false-positive diagnosis. Three-dimensional papillary clusters in a breast aspirate may be seen in a broad spectrum of conditions, namely papillary hyperplasia in fibrocystic disease, duct papilloma, papillary or micropapillary ductal carcinoma and pseudopapillary pattern in invasive ductal carcinoma (not otherwise specified). This wide variation prompted us to analyse papillary lesions in cytological smears and histopathology and correlate them. This study was also carried out with the aim of evaluating the utility of individual morphological features in offering a more precise cytodiagnosis in papillary lesions.

## Materials and Methods

Cases reported as papillary lesions on nipple discharge and fine needle aspiration cytology (FNAC) and on histopathology during the past two years were included in the study. The cytology slides [May-Grünwald-Giemsa (MGG) and hematoxylin and eosin (H and E)-stained slides] and H and E-stained histopathology slides were retrieved from the files and a retrospective comparative analysis was carried out. Immunohistochemistry for smooth muscle actin and S 100 protein was performed on paraffin-embedded sections wherever needed. A subjective assessment of the following morphological features, namely, the cellular yield, presence of three-dimensional papillary clusters, stromal bare nuclei, presence of cyst macrophages and cellular atypia, was carried out on cytosmears [Table 1].

**Table 1:** Assessment criteria for evaluating cytosmears

Parameters	Assessment and quantification	
Cellularity	Low	+
	Moderate	++
	High	+++
3-dimensional clusters	Present	+
	Absent	++
Cyst macrophages	Occasional	+
	Few	++
	Numerous	+++
Stromal bare nuclei	Occasional	+
	Few	++
	Numerous	+++
Cellular atypia	Low	+
	Moderate	++
	High	+++

The results were correlated with the histological diagnosis. The pitfalls leading to false-positive and false-negative results were analysed.

## Results

**Figure 1:**
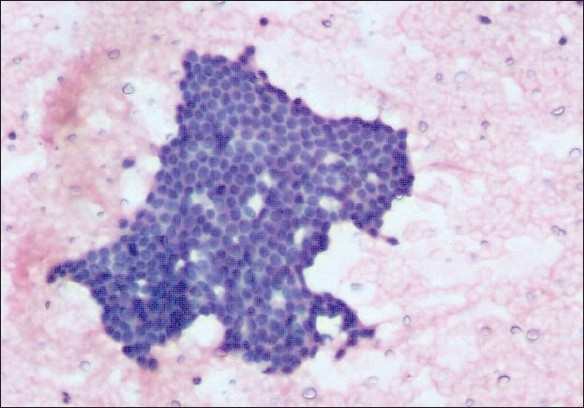
Atypical papilloma reported as benign proliferative breast disease on cytology shows moderately cellular cytosmear with ductal cells arranged in monolayered cohesive sheets and few bare nuclei (H and E, × 100)

**Figure 2:**
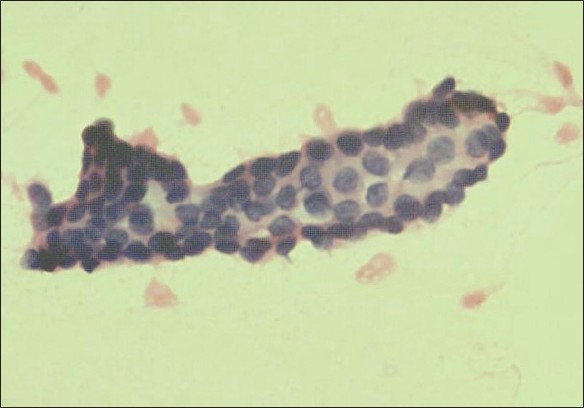
False-negative cytosmear with poor cellularity showing papillary structures (H and E, × 200)

On retrospective analysis of cytology and histopathology slides, 14 cases of papillary lesions were identified [Table 2]. The age group ranged from 27 years to 62 years. The number of cases reported was more in the fifth decade. Seven of the 14 cases were diagnosed as malignant lesions on histopathology [Table 2]. Thirty-six per cent of the cases were found to be true positive, 43% false negative [Figures 1 and 2] and 21% false positive with a sensitivity of 42% for papillary lesions. Cellularity was high in four of the malignant lesions and in three of the benign lesions irrespective of the papillary nature [Table 2]. Three-dimensional papillary clusters were seen in four of the nine papillary lesions and three of the five nonpapillary lesions. Eight of nine papillary lesions showed cyst macrophages. Stromal bare nuclei were seen in three of four malignant papillary lesions. However, the number of stromal bare nuclei was less compared with benign lesions. None of the malignant nonpapillary lesions showed stromal bare nuclei [Table 2]. Three of four malignant papillary lesions showed cellular atypia ranging from a mild to a severe degree [Table 2]. Presence of three-dimensional clusters and cyst macrophages was analysed to determine their sensitivity and specificity for identifying papillary lesions [Table 3a]. Sensitivity and specificity were also derived for the presence of bare nuclei and nuclear atypia in benign and malignant papillary lesions [Table 3b].

**Table 2:** Assessment of the various morphological features on cytosmears

Age in years	Cytodiagnosis	Cellularily	3D clusters	Cyst macrophages	Bare nuclei	Atypia	Final diagnosis on histopathology
48	Papillary lesion	+++	−	+	−	+	Invasive ductal carcinoma
52	Papillary carcinoma	+++	+	−	−	+++	Papillary carcinoma
38	Duct papillomatosis	+++	+	+	++	−	Fibroadenoma
42	Papillary neoplasm	+++	+	+	−	+	Invasive ductal carcinoma with papillary areas
60	Papillary neoplasm	++	+	+	−	−	Intaductal papilloma
53	Fibrocystic disease with epitheliosis	++	−	+++	++	−	Duct papilloma
34	Papillary neoplasm	+++	+	+	−	++	Duct papilloma
52	Fibrocystic disease with epitheliosis	++	−	++	+	−	Invasive papillary carcinoma
40	Benign proliferative breast disease	+++	−	+	+	−	Atypical papilloma
27	Fibroadenoma	++	−	++	++	−	Fibrocystic disease with papillomatosis
60	Proliferative breast diasease with apocrine change	+++	−	+	−	+	Intaductal papillary carcinoma
62	Proliferative breast disease with papillary structures	++	+	−	−	−	Invasive ductal carcinoma with desmoplasia
35	Cystic papillary neoplasm	++	+	+	+	++	Intracystic papillary carcinoma with invasion
37	Proliferative breast disease	++	−	+	+	+	Intraductal papilloma

**Table 3a:** Assessment of sensitivity and specificity for papillary lesions on cytosmears

	3-dimensional clusters	Cyst macrophages
Sensitivity	71.4%	83.3%
Specificity	85.7%	50%

**Table 3b:** Assessment of sensitivity for benign and malignant papillary lesions

	Bare nuclei	Nuclear atypia
Sensitivity	60%	66.6%
Specificity	40%	40%

## Discussion

Papillary lesions of breast encompass a wide spectrum of benign and malignant entities constituting <2% of all breast carcinomas.[1] These lesions have a variable clinical and radiological presentation, causing diagnostic difficulties. Papillary lesions present clinically as a palpable mass or nipple discharge and, at times, these features may not be evident. Mammogram may show multiple bilateral lesions of varying sizes with or without microcalcifications. Ultrasound of these lesions may show a complex intracystic lesion or a homogenous solid lesion.[2] However, even if the lesion is identifiable on radiologic imaging, such detection is neither sensitive nor enough to accurately differentiate malignant and benign papillary tumors.[2]

The cytodiagnosis of papillary neoplasms of the breast continues to be a gray zone. Interpretation errors are due to pseudopapillary structures and high cellularity encountered in certain nonpapillary lesions. This retrospective study was carried out in an attempt to identify more reliable cytological criteria for the diagnosis of papillary lesions of breast.

The overall sensitivity of cytological diagnosis of papillary breast lesions was 42% in our study. This is in concordance with the low diagnostic accuracy reported for these lesions in the literature.[3] Assessment of cellular yield was not found to be a useful parameter in distinguishing papillary from nonpapillary lesions. Malignant lesions showed a high cellular yield irrespective of their papillary or nonpapillary nature. Gomez *et al*.,[4] in their study, found abundant cellular material in papillary carcinoma and relatively less material in papilloma, which was in concordance with our study. Dowson *et al*.,[5] in their analysis of papillary lesions, found increased cellularity and presence of single cells in the background helpful in distinguishing papillary carcinomas from benign papillomas. Increased cellularity in malignant lesions was a common finding in both the above-mentioned studies, which was also noted in our study.

In our study, although three-dimensional clusters on cytosmears had a high specificity for recognizing papillary lesions, we found pseudopapillary structures in fibroadenoma and invasive ductal carcinoma NOS similar to Michael *et al*.[6] Cyst macrophages were identified consistently in papillary lesions and were more in number with a high sensitivity. This was in concordance with the findings of Jeffry *et al*.[7] Stromal bare nuclei and nuclear atypia showed a low sensitivity and specificity in distinguishing benign and malignant papillary lesions in our study as against the findings of Nayar *et al*.[8] Hence, these parameters may not be of much value in differentiating benign and malignant papillary lesions, as there is a considerable overlap in the degree of cellular atypia in these lesions. Kumar *et al*.,[9] in their study, reviewed nine cases of invasive papillary carcinoma and found eosinophilic bipolar cytoplasmic granules. This feature was not seen in our cases.

In the absence of reliable cytomorphological criteria to distinguish papillary from benign and malignant nonpapillary lesions, immunohistochemistry for smooth muscle actin was found to be useful in identifying the myoepithelial cells pointing towards a benign nature.[10,11] A more elaborate study including more number of cases and immunohistochemical work-up may provide a better understanding of this complex problem.

The diagnostic dilemma in papillary lesions of the breast is just not limited to clinical, radiological and cytological features but also occurs in core biopsies where under-sampling can be a problem. Immunohistochemical work-up to identify myoepithelial cells is useful in distinguishing well-differentiated papillary carcinoma from atypical papilloma. Distinguishing papillary carcinoma from other papillary lesions of the breast is extremely important, therefore excision biopsy of the entire lesion is necessary in all suspected papillary lesions.

An unequivocal cytological diagnosis of papillary carcinoma is known to be an extremely difficult task as clear distinction between benign and malignant lesions cannot be made on cytology alone. To conclude, cytomorphological features alone are not sufficient for a confident diagnosis of papillary lesions of the breast. Histopathological examination plays a pivotal role in the final diagnosis of these lesions.
